# Assessment of Treatment Outcomes and Associated Factors Among Pediatric Patients With Burkitt Lymphoma at Kenyatta National Hospital

**DOI:** 10.1002/cnr2.70112

**Published:** 2025-01-13

**Authors:** Divya Kumari Toor, Amsalu Degu, Peter N. Karimi

**Affiliations:** ^1^ School of Pharmacy and Health Sciences, Department of Pharmaceutics and Pharmacy Practice United States International University‐Africa Nairobi Kenya; ^2^ Department of Pharmacology, Clinical Pharmacy and Pharmacy Practices, Faculty of Health Sciences University of Nairobi Nairobi Kenya

**Keywords:** Burkitt lymphoma, Kenyatta National Hospital, predictors, treatment outcomes

## Abstract

**Background:**

In developing countries, the treatment outcomes of Burkitt lymphoma are poor due to the poorly equipped healthcare systems. In addition, there is limited comprehensive data within the African continent, including Kenya, about the outcomes of treatment for this cancer.

**Aims:**

To assess treatment outcomes and variables associated with an increased risk of death from disease progression or treatment‐related toxicities among Burkitt lymphoma pediatric patients at the Kenyatta National Hospital (KNH).

**Methods and Results:**

A retrospective one‐arm cohort study was conducted to examine the treatment outcomes of pediatric patients with Burkitt lymphoma. All eligible Burkitt lymphoma pediatric patients treated between January 1, 2016 and December 31, 2022 were included. The patients were retrospectively monitored from the initial cancer diagnosis until either death or the last follow‐up appointment visit in the facility. Data analysis of factors associated with treatment and disease progression‐related death was carried out using the SPSS version 29.0 software. Kaplan–Meier survival and Cox regression analyses were employed to determine the survival time and predictors of mortality, respectively. The median age of the patients at diagnosis was 6 years (range: 3–13 years). The majority of patients were diagnosed with Stage IV disease accounting for 46.7% of all patients. Of the 75 patients studied, 24% (18) of them were died. The 5‐year overall survival rate was 70%, and most patients had stable disease during the follow‐up period. Patients with Stage IV disease who were treated with full‐fuse chemotherapy were 19.2 (AHR = 19.2, 95% CI = 5.2–48.5, *p* < 0.001) and 7.4 times (AHR = 7.4, 95% CI = 2.2–19.9, *p* = 0.003) more hazard of dying as compared to patients without metastasis and received a combination of radiation and reduced‐dose chemotherapy, respectively. However, the age, gender, stage of cancer, histological type of cancer, and co‐morbidity were not significant predictors of survival. Because of the retrospective nature of the study design, the data accuracy relied on the proper documentation of medical records in the study setting.

**Conclusion:**

The 5‐year overall survival rate among pediatric burkitt's lymphoma patients was above average as compared to other African countries. Most patients had reduced tumor size and stable disease during the follow‐up period. Metastases and full‐fuse chemotherapy were significant predictors of mortality.

## 1 Background

Burkitt's lymphoma (BL) is an aggressive B‐cell lymphoma associated with chromosomal translocations that result in the overexpression of the oncogene, C‐MYC [1]. Endemic BL is most commonly associated with viruses such as Epstein–Barr virus (EBV), and human immunodeficiency virus (HIV).

The yearly incidence in children under the age of 18 is around six cases per 100 000 children, with a median age at diagnosis of 6 years [2, 3]. BL is more prevalent in males than in females, presenting with a ratio of 4:1 [4]. The incidence of BL varies widely by geographic location, specifically when comparing endemic versus sporadic BL [5, 6, 7].

The three subtypes of BL are endemic, sporadic, and immunodeficiency‐related, with the endemic type being more common in the African region. The endemic BL is very common in *Plasmodium falciparum* malaria endemic regions in sub‐Saharan Africa [6]. In Africa, BL comprises nearly 50% of non‐Hodgkin lymphoma in the pediatric population [7]. Previous studies reported a high prevalence of BL in Kenya [7, 8]. Recent studies in Kenya showed a 22% mortality rate among pediatric patients diagnosed with endemic BL due to limitations in access to care and the availability of supportive care [2].

In developing countries, the treatment outcomes of this malignancy are suboptimal due to the poorly equipped healthcare systems which contribute to late diagnosis [9]. The gap in the treatment outcomes is due to the challenges faced by poorly equipped healthcare systems, leading to higher mortality and low survival compared to high‐income countries [10, 11]. In addition, lack of awareness of the disease, late presentations, financial constraints, and treatment‐related toxicities are the major contributing factors to suboptimal treatment outcomes [9, 11]. Hence, this study purposed to assess the treatment outcomes among pediatric BL patients at the Kenyatta National Hospital (KNH).

## 2 Methods

### 2.1 Study Design, Setting, and Period

A retrospective one‐arm cohort study was carried out to investigate the treatment outcome and associated factors among pediatric BL patients at KNH between 2016 and 2022. This study was conducted at KNH for 6 months (December 1, 2022 to March 31, 2023). The facility is the largest referral and teaching hospital in Kenya and has a well‐established cancer unit that handles cases from the entire country and neighboring countries.

### 2.2 Study Population

The study population consisted of all eligible pediatric patients diagnosed with BL who were treated at the KNH between 2016 and 2022.

## 3 Eligibility Criteria

### 3.1 Inclusion Criteria

Patients aged up to 12 years with a confirmed diagnosis of BL treated from January 1, 2016 to December 31, 2022 with comprehensive medical records of the treatment regimens, stage of cancer, and diagnosis.

### 3.2 Exclusion Criteria

Patients who had gaps in their medical records on their treatment regimen, cancer diagnosis, and cancer stage.

### 3.3 Sample Size and Sampling Technique

All pediatric patients diagnosed with BL who met the inclusion criteria and had received treatment at the KNH between the years 2016 and 2022 comprised the study's sample size. In this study, 75 eligible patients were included.

### 3.4 Data Collection Procedures

Data were collected from patients' medical records using a data abstraction tool. The tool included data on socio‐demographics, clinical characteristics, and parameters measuring survival outcomes, such as mortality, remission status after treatment, tumor size, and CD‐20 and CD‐79 levels. These tumor markers (CD‐20 and CD‐79) were used to assess treatment response among BL patients. Reduction in the tumor marker levels suggested optimal response in these patients. The clinical characteristics of the tool comprised the histologic types, co‐morbidities, stage of cancer, and treatment modalities. The data abstraction tool was designed based on previous studies [12, 13, 14, 15, 16].

Prior to using the data collection tool in the main study, a pretest was carried out on 5% of the total sample size. Necessary adjustments were made based on the pretest results. After sourcing the medical records of the patients from the KNH Health Information Department, the records were reviewed to collect socio‐demographics, clinical information, survival time, and response to therapy. Mortality, survival time, and treatment response were reported as outcomes.

### 3.5 Data Analysis

The data collected were evaluated using the SPSS version 29.0 software. Kaplan–Meier survival analysis was carried out to examine the survival time. Bivariable and multivariable Cox proportional hazard regression analyses were used to investigate the predictors of survival. The variables included were age, stage of cancer, histological type of cancer, comorbidity, distant metastasis, and the type of treatment regimen. The results were presented using percentages and frequencies.

## 4 Results

### 4.1 Socio‐Demographics

The mean age and median follow‐up time were 6.96 ± 3.03 years (range: 3–13 years) and 10.7 months, respectively. Most of the patients were males and accounted for 73.3%. The majority (88%) of them were in primary school. Of all the patients, 84% did not have a family history of cancer (Table 1).

**TABLE 1:** Socio‐demographic characteristics.

Variable	Frequency	%
Gender		
Female	20	26.7
Male	55	73.3
Age (years)		
< 5 years	31	40.8
≥ 5 years	44	57.9
Level of education		
Not enrolled in school	9	12.0
Primary	66	88.0
Family history		
No	63	84.0
Yes	12	16.0

### 4.2 Clinical Characteristics and Treatment Modalities

Endemic BL was the most prevalent (62.7%) histological type followed by sporadic BL (33.3%) and immunodeficiency‐associated BL (4.0%). Most patients were diagnosed with Stage IV disease accounting for 46.7%. The most prevalent comorbidity was diabetes mellitus type 1 accounting for 9.3% of all patients (Table 2).

**TABLE 2:** Clinical characteristics of the study participants.

Variable	Frequency	%
Stage of cancer		
Stage I	8	10.7
Stage II	16	21.3
Stage III	16	21.3
Stage IV	35	46.7
Histology of Burkitt's lymphoma		
Endemic	47	62.7
Sporadic	25	33.3
Immunodeficiency associated	3	4.0
Comorbidity		
Absent	61	81.3
Present	14	18.7
Type of comorbidity		
Diabetes mellitus type I	7	9.3
HIV	3	4.0
Hypertension	2	2.7
Arrhythmia	1	1.3
HIV and diabetes mellitus type I	1	1.3

Abbreviation: HIV, human immunodeficiency virus.

The treatment regimens used were chemotherapy (43.4%) as well as chemotherapy and surgery (27.6%). Most patients (53.3%) underwent 1–15 cycles of chemotherapy (Table 3).

**TABLE 3:** Treatment regimen of multiple myeloma patients.

Variable	Frequency	%
Treatment modalities		
Chemotherapy	33	43.4
Chemotherapy + surgery	21	27.6
Chemotherapy + surgery + radiotherapy	15	19.7
Chemotherapy + radiotherapy	5	6.6
Chemotherapy + surgery + physiotherapy	1	1.3
Type of chemotherapy regimen		
CHOP	52	68.4
ICE	9	11.8
COPADM	7	9.2
CYM	2	2.6
M‐CHOP	2	2.6
R‐CHOP	2	2.6
CVF	1	1.3
Number of cycles		
1–15	40	53.3
16–31	21	28.0
≥ 31	14	18.7

Abbreviations: CHOP, cyclophosphamide + doxorubicin + vincristine + prednisolone; COPADM, cyclophosphamide + vincristine + prednisolone + daunorubicin + methotrexate; CVF, cyclophosphamide + vinorelbine + 5‐fluorouracil; CYM, cytarabine + methotrexate; ICE, ifosfamide + carboplatin + etoposide; M‐CHOP, methotrexate + cyclophosphamide + doxorubicin + vincristine + prednisolone; R‐CHOP, rituximab + cyclophosphamide + doxorubicin + vincristine + prednisolone.

### 4.3 Treatment Outcomes of BL in Pediatric Patients

Most patients (69.3%) had no metastases, but 30.7% had metastasis during the follow‐up period. About two‐thirds (76%) of patients were censored, and 24% were deceased. For about half of the patients, the CD‐20 (44%) and CD‐79 (45.3%) levels significantly decreased. This suggested most patients had optimal responses due to the reduction of these tumor markers after treatment. More than half (57.3%) had decreased tumor size while two‐thirds of the patients (34.7%) had stable disease (Table 4).

**TABLE 4:** Treatment outcomes of the study participants.

Variable	Frequency	%
Tumor size after treatment		
Decreased	43	57.3
Progressed	27	36.0
No change	5	6.7
Metastasis during the follow‐up period		
No	52	69.3
Yes	23	30.7
Patient status after treatment		
Censored	57	76.0
Dead	18	24.0
Remission status of the patient		
Complete remission	12	15.8
Partial remission	8	10.5
Disease progressed	8	10.5
Stable disease	26	34.2
No response	3	3.9
CD‐20 level in the last follow‐up period		
No change	14	18.7
Significantly increased	28	37.3
Significantly decreased	33	44.0
CD‐79 level in the last follow‐up period		
No change	14	18.7
Significantly increased	27	36.0
Significantly decreased	34	45.3

The mean‐cancer‐specific survival time was 13.1 ± 10.9 months. The metastasis‐free survival and cancer‐specific survival after metastases were 1.6 ± 2.2 months and 3.9 ± 5.9 months, respectively. The 5‐year survival rate of pediatric patients diagnosed with BL was 70%. The log‐rank test depicted that there was a significant difference in the mean survival time among the different age groups, co‐morbidity, histological type of cancer, stage of disease, and treatment modalities. The mean survival time of co‐morbid patients was 10.4 ± 1.8 months, while 48.8 ± 3.4 months in patients without co‐morbid conditions. Immunodeficiency‐associated types of BL had the shortest mean survival (8.3 ± 3.2 months) than the other histological types. The mean survival time of those patients with early‐stage cancer was 46.5 ± 7.3 months, whereas for those with advanced‐stage cancer, it was 33.9 ± 4.1 months. Chemotherapy‐treated patients had the highest mean survival time (42.3 ± 7.3 months) as compared to combination treatment modalities (Table 5).

**TABLE 5:** Mean survival time among the study participants.

Variable	Mean survival time (in months) ± standard error of the mean (95% CI)	Log–rank test (*p*)
Age		0.006*
< 5 years	35.7 ± 1.6 (32.5–38.8)	
≥ 5 years	34.1 ± 4.9 (24.5–43.7)	
Gender		0.613
Male	5.7 ± 0.67 (4.33–6.99)	
Female	3.9 ± 0.78 (2.43–5.52)	
Co‐morbidity		< 0.001*
Present	10.4 ± 1.8 (6.9–13.9)	
Absent	48.8 ± 3.4 (42.1–55.4)	
Stage of cancer		0.013*
Early stage (I and II)	46.5 ± 7.3 (32.2–60.9)	
Advanced stage (III and IV)	33.9 ± 4.1 (25.9–41.9)	
Histological types of cancer		0.010*
Endemic	42.1 ± 4.6 (33.2–51.1)	
Sporadic	32.2 ± 2.7 (27.0–37.4)	
Immunodeficiency‐associated	8.3 ± 3.2 (2.1–14.6)	
Type of treatment regimen		< 0.001*
Chemotherapy	42.3 ± 7.3 (28.0–56.6)	
Combination therapy	37.1 ± 3.9 (29.5–44.7)	

* Statistically significant with *p* value < 0.05.

### 4.4 Predictors of Mortality Among the Study Participants

Patients who had metastasis and treated with full fuse chemotherapy were 19.2 (AHR = 19.2, 95% CI = 5.2–48.5, *p* < 0.001) and 7.4 times (AHR = 7.4, 95% CI = 2.2–19.9, *p* = 0.003) more hazard of dying as compared to patients without metastasis and received combination of radiation and reduced dose chemotherapy, respectively. However, the other parameters, such as age, gender, stage of cancer, histological type of cancer, and co‐morbidity, were not significant predictors of survival (Table 6).

**TABLE 6:** Predictors of mortality among pediatric patients with Burkitt lymphoma.

Variable	Bivariable analysis		Multivariable analysis	
	CHR (95% CI)	*p*	AHR (95% CI)	*p*
Gender				
Male	1		1	0.220
Female	0.7 (0.28–1.9)		0.5 (0.2–1.9)	0.230
Age				
< 5 years	1		1	
≥ 5 years	1.4 (1.1–13.4)	0.031*	0.9 (0.2–3.5)	0.843
Stage of cancer				
Early stage (I and II)	1		1	
Advanced stage (III and IV)	1.7 (1.2–23.1)	0.028*	1.5 (0.2–10.3)	0.678
Histological type of cancer				
Immunodeficiency‐associated	1		1	
Endemic	0.32 (0.07–1.4)	0.136	1.4 (0.3–6.7)	0.638
Sporadic	0.26 (0.05–1.3)	0.546	0.9 (0.1–5.6)	0.874
Co‐morbidity				
Absent	1		1	
Present	1.4 (0.9–0.6)	0.03*	1.2 (0.4–3.7)	0.742
Metastasis during the follow‐up period				
No	1		1	
Yes	22.0 (5.0–67.7)	< 0.001*	19.2 (5.2–48.5)	< 0.001*
Type of treatment regimen				
Combination of radiation and reduced dose chemotherapy	1		1	
Full fuse chemotherapy	19.0 (1.6–21.6)	0.005*	7.4 (2.2–19.9)	0.003*

Abbreviations: AHR, adjusted hazard ratio; CHR, crude hazard ratio.

* Statistically significant with *p* value < 0.05.

## 5 Discussion

Previous studies in the United States and Brazil reported improved survival among pediatric BL patients [17, 18, 19]. In Africa, the survival of the pediatric population diagnosed with BL is poor despite the lack of sufficient data [11, 20, 21, 22]. Hence, this retrospective study purposed to assess the treatment outcomes and their predictors among pediatric BL patients at KNH.

The results of this study revealed that the majority of the patients were above the age of 5 years, with a mean age of 6.96 ± 3.03 years. A previous study found that, in Kenya and other African regions, the mean age was 6 years at diagnosis [8, 23]. This study also showed that male patients were more than female patients. This observation concurs with other studies that show male predominance [8, 24, 25]. This could be probably due to the predominance of EBV infection and the presence of a more unstable genetic composition that does not withstand genetic injury in males as compared to females [26, 27].

In Africa, EBV and malaria are thought to be potential co‐factors in both sporadic and endemic types of BL [28]. BL linked to immunodeficiency is more prevalent in patients infected with HIV. Endemic BL is usually associated with EBV infection and malarial parasites [4]. In this study, the endemic type was the most prevalent histological type, followed by the sporadic type. This could be due to the high prevalence of malaria in our setting, which can weaken the immune response to the EBV infection, allowing it to alter infected B cells to become malignant [29].

In this study, some of the patients had comorbidities, and type 1 diabetes mellitus was the most common type, followed by hypertension. Diabetes has been observed as one of the comorbidities found in pediatric BL patients elsewhere [30]. A study conducted in Germany pointed out that endocrine tissue was damaged by displacement and destruction due to the tumor, which led to islet cell injury [30]. Having a comorbidity and being diagnosed with an advanced stage of cancer were associated with considerably worsening survival [30]. The role comorbidities play in survival may be related to an increased risk of treatment‐related complications [30].

In this study, most patients had a decrease in tumor size following therapy, whereas others had an increase in the size of the tumor and a few experienced a progression in the disease. The disease progression and increase in tumor size may be due to late diagnosis, because most patients were diagnosed in the advanced stage. Previous studies conducted in Lebanon reported that treatment failures in patients with Stages III and IV were due to tumor lysis, intracranial hemorrhage, and progressive disease [31]. A study in South Africa reported that 47 patients presented with advanced‐stage disease [22].

The 5‐year survival rate was 70% in our setting. This finding agrees with a couple of studies conducted in various countries, such as Central Brazil, South Africa, Western Kenya, Uganda, and Malawi, which reported 5‐year survival rates from 50.5% to 70% [2, 11, 19, 22]. In contrast, other studies showed higher survival rates. For instance, the United States and Europe reported a 5‐year survival rate of 90% [17, 32]. Other studies conducted in Sub‐Saharan Africa showed lower 5‐year survival rates ranging from 30% to 50% which has not changed from the 1970s era while another reported a survival rate of 60% [20, 21]. The variation in the overall survival rates is possibly linked to the difference in the stage of disease, level of care, early screening, and co‐morbidities [2, 33, 34]. Children with BL with Stage IV disease have poor outcomes [17]. Due to the high tumor growth rate, patients often present with advanced disease. Nearly, 70%–90% of patients were diagnosed with widespread disease [19, 35].

Metastases and the full‐fuse chemotherapy treatment regimen were the important factors that impacted survival. This finding concurs with studies conducted in the United States and Uganda [11, 17]. Hence, the exploration of less toxic or more targeted chemotherapy regimens and immunotherapies that may offer improved outcomes with fewer side effects should be considered, particularly for advanced disease patients [21, 36, 37]. In addition, close monitoring and follow‐up are highly recommended among patients with metastatic disease and full‐intensity chemotherapy‐treated patients [36]. Implementation of early detection of metastases through more frequent monitoring and advanced imaging techniques and early initiation of optimal treatment regimens is recommended to improve survival outcomes among BL patients [38, 39].

Due to the little accessible information in our context, more research on BL is required, specifically on the importance of CD‐20 and CD‐79 as helpful tools for outcome‐measuring metrics. Further investigation should be conducted to examine dosing schedules, treatment compliance, toxicity management, and support systems during full‐fuse chemotherapy and in patients with metastatic disease.

## 6 Limitations of the Study

The study was retrospective, and then the data accuracy relied on the proper documentation of medical records in the study setting. Some records were incomplete and were consequently excluded, leading to a smaller sample size.

## 7 Conclusion

The 5‐year overall survival rate among pediatric BL patients was above average as compared to other African countries. Most patients had reduced tumor size and stable disease during the follow‐up period. Metastases and full‐fuse chemotherapy were the statistically significant predictors of mortality.

## Author Contributions

**Divya Kumari Toor:** conceptualization, investigation, funding acquisition, writing – original draft, methodology, writing – review and editing, software, project administration, formal analysis, validation, visualization, data curation, and resources. **Amsalu Degu:** data curation, methodology, conceptualization, investigation, funding acquisition, writing – original draft, validation, visualization, writing – review and editing, software, project administration, formal analysis, resources, and supervision. **Peter N. Karimi:** conceptualization, data curation, supervision, resources, software, methodology, visualization, validation, investigation, funding acquisition, writing – original draft, formal analysis, project administration, writing – review and editing.

## Ethics Statement

The study was approved by the University of Nairobi/Kenyatta National Hospital Ethics and Research Committee (Approval number: KNH‐ERC/UA/361). The confidentiality of patients' information was maintained by not recording patient identifiers such as the name and address of the study participants.

## Consent

A waiver of informed consent was granted by the Ethics Committee because the study was retrospective and did not involve the patients directly.

## Conflicts of Interest

The authors declare no conflicts of interest.

## Data Availability

The data that support the findings of this study are available from the corresponding author upon reasonable request.
